# *Roseburia*-Associated Gut–Brain Axis Alterations in Relapsing–Remitting Multiple Sclerosis: Evidence from a Household-Matched Case–Control Study

**DOI:** 10.3390/nu18071117

**Published:** 2026-03-31

**Authors:** Alen Zollo, Matteo Domenico Marsiglia, Andrea Corona, Emerenziana Ottaviano, Maria Laura Terzi Mazzieri, Alessandra Mingione, Silvia Ancona, Alberto Priori, Elisa Borghi, Filippo Martinelli-Boneschi

**Affiliations:** 1 Department of Health Sciences, University of Milan, 20142 Milan, Italy; azollo@uninsubria.it (A.Z.); matteo.marsiglia@unimi.it (M.D.M.); andrea.corona@unimi.it (A.C.); emerenziana.ottaviano@unimi.it (E.O.); marialaura.terzimazzieri@studenti.unimi.it (M.L.T.M.); alessandra.mingione@unimi.it (A.M.); silvia.ancona@unimi.it (S.A.); alberto.priori@unimi.it (A.P.); filippo.martinelli@unimi.it (M.-B.F.); 2 CRC “Aldo Ravelli” for Experimental Brain Therapeutics, University of Milan, 20142 Milan, Italy; 3 Clinical Neurology Unit, San Paolo Hospital, ASST Santi Paolo e Carlo, 20142 Milan, Italy

**Keywords:** multiple sclerosis, microbiota–gut–brain axis, *Roseburia*, clinical severity

## Abstract

**Background/Objectives:** Gut microbiota (GM) dysbiosis has been implicated in multiple sclerosis (MS) pathogenesis, influencing inflammation and neurodegeneration, but findings remain inconsistent due to environmental and methodological variability. This study aimed to identify possible microbial biomarkers of MS status and disease severity by profiling gut microbiota and short-chain fatty acid (SCFA) patterns in people with relapsing–remitting MS (pwRRMS), using household-matched healthy controls (HC) to minimize environmental variability. **Methods:** Twenty-four pwRRMS and their respective household-matched healthy controls (HC) were enrolled, with dietary and lifestyle habits monitored. GM composition was assessed by 16S rRNA gene sequencing, and fecal SCFAs were quantified using gas chromatography–mass spectrometry. PwRRMS were stratified by Expanded Disability Status Scale (EDSS) and Multiple Sclerosis Severity Score (MSSS). **Results:** β-diversity did not differ between groups. However, α-diversity was significantly reduced in pwRRMS, particularly in those with greater disability. Reduced diversity was associated with lower abundance of butyrate-producing genera (*Roseburia*, *Faecalibacterium*, *Coprococcus*) and enrichment of *Oscillibacter* and *UBA1819*, alongside a downward trend in fecal butyrate and propionate levels. **Conclusions:** RRMS and greater disease severity are associated with gut microbial alterations characterized by reduced SCFA-producing bacteria. Despite limitations including small sample size and sex imbalance, the household-matched design strengthens internal validity. Our findings highlight the potential of targeting the gut microbiota, an accessible compartment within the gut–brain axis, for MS management.

## 1. Introduction

Multiple sclerosis (MS) is a complex autoimmune disease of the central nervous system influenced by both genetic and environmental factors [1]. With growing support for the holobiont theory, which views humans as organisms composed of mammalian cells together with their resident microbiome, the role of commensal microorganisms in various complex diseases has gained significant attention [2]. Notably, the gut–brain axis, a bidirectional communication pathway where gut microbes communicate with the central nervous system (CNS), has been shown to influence various aspects of physiological and neurological function [3]. Gut microbiota (GM) changes have been linked to seizure occurrence, autism, and other neurological disorders [4].

GM composition in persons living with MS (pwMS) has also been investigated, with several genera identified as either contributing to or protecting against the disease [5]. Beyond the disease itself, the gut microbiota is shaped by a wide range of factors, including diet, genetics, and lifestyle. This complexity may help to explain the inconsistent findings reported across MS cohorts regarding microbial composition. Variations in study design, population characteristics, and environmental influences likely contribute to the discrepancies observed in the alterations of microbial taxa. Indeed, diet is a major determinant of microbial composition [6], along with genetics [7], geographic location, and lifestyle-related elements [6,8].

To address these gaps, we aimed at identifying potential microbial biomarkers associated with MS status and disease severity by profiling GM composition and fecal short-chain fatty acid (SCFA) levels in individuals with relapsing–remitting MS (pwRRMS). To minimize environmental confounding, we characterized the gut microbiota of 24 pwRRMS, each matched with a cohabiting healthy control (HC). All participants resided in the same geographical area, dietary intake was monitored using a food diary, and lifestyle factors were assessed through a structured questionnaire to account for additional potential confounders. This design allowed us to explore whether specific microbial and SCFA patterns distinguish pwRRMS from HC and whether these features are associated with clinical measures of disease severity and disease disability.

## 2. Materials and Methods

### 2.1. Cohort Recruitment and Specimen Collection

Persons with relapsing–remitting multiple sclerosis (pwRRMS) and their cohabiting HC were recruited at ASST Santi Paolo e Carlo, San Paolo Hospital, and Ospedale Maggiore Policlinico of Milan between 2022 and 2023. Inclusion criteria were an MS diagnosis according to the revised McDonald criteria [1], a relapsing–remitting disease course, no relapses in the previous three months, and the absence of other autoimmune, neurological, and gastrointestinal disorders. PwRRMS were excluded if they had initiated a new treatment within the previous three months. Participants who had received antibiotics in the previous three months, corticosteroids in the previous month, or had undergone intestinal surgery were excluded.

pwRRMS were grouped into three EDSS disability categories: low (EDSS 0–2), medium (EDSS 2.5–4), and high (EDSS > 4) [9]. MSSSs were categorized as follows: low (≤2.5; indicating slow disease progression), medium (2.5–5.0; moderate progression), and high (>5.0; rapid progression) [10].

All participants who provided written informed consent received a stool sample self-collection kit (OMNIgene•GUT OM-200, DNA Genotek Inc., Ottawa, ON, Canada). Samples were self-collected, stored at room temperature, and hand-delivered by patients at the clinical evaluation. Stools were then stored at −80 °C in the laboratory for further processing.

Each participant filled out a seven-day food diary encompassing both weekly and weekend days, following researcher guidelines before stool sample collection. The dietary records were processed using commercially available software (MetaDieta 4.4, METEDA srl., Rome, Italy). A questionnaire with information on lifestyle, clinical records, and socio-demographic data was administered.

### 2.2. 16S rRNA Sequencing and Fecal Short-Chain Fatty Acid Quantification

DNA was extracted using the QIAamp Powerfecal pro DNA kit (Qiagen, Hilden, Germany) as per manufacturer instructions and quantified via Qubit^®^ (Thermo Fisher Scientific, Waltham, MA, USA). The sequencing of the V3–V4 hypervariable regions of the bacterial 16S rRNA gene was performed in service by Macrogen (Seoul, Republic of Korea), according to the Illumina 16S Metagenomic Sequencing (Illumina, San Diego, CA, USA).

Short-chain fatty acids (SCFAs) were extracted as follows. 300 µL of the stool-stabilizing buffer mixture was weighed and combined with 700 µL of water, 200 µL of pure orthophosphoric acid (85%), and 100 µL of 2-ethylbutyric acid (3.3 mM). 500 µL of a diethyl ether/heptane solution was added, and the organic phase was separated after centrifugation. Quantification of acetic, propionic, isobutyric, butyric, and isovaleric acids was carried out using gas chromatography-mass spectrometry (GC-MS) with an Agilent 8860 GC system coupled to a 5977C MSD detector. A DB-WAX Ultra Inert capillary column (30 m, 0.25 mm, 0.25 µm, Agilent Technologies, Santa Clara, CA, USA) was used for separation. Compound identification was achieved by comparing retention times and mass spectra with those of pure standards. Data processing was performed with MassHunter software 12.1 (Agilent Technologies). Results are expressed as nmol of SCFA in mg of stool-stabilizing buffer mixture. Stool samples were collected directly into the stabilizing buffer, and the initial mass of fecal material was not recorded separately prior to processing, precluding expression of SCFA concentrations in standard units (e.g., µmol/g feces). SCFA concentrations were therefore normalized to the total mass of the stool–buffer mixture to ensure consistency across samples within the study.

### 2.3. Statistical Analysis

Raw 16S rRNA gene sequences were processed using the DADA2 pipeline in R (v1.30.0), which included quality filtering, trimming, denoising, and chimera removal. Taxonomic classification was performed by aligning amplicon sequence variants (ASVs) to the SILVA reference database [11]. Secondary analyses were conducted using MicrobiomeAnalyst 3.0 [12], where features with low counts or low variance were filtered out. Data were normalized using total sum scaling without rarefaction or transformation, enabling relative abundance comparisons.

Microbial diversity was assessed using alpha-diversity indices (Chao1, Fisher’s, Shannon, Simpson, and Observed Species) and beta-diversity based on Bray–Curtis dissimilarity, calculated from the ASV table. Relative abundances of taxa are reported as percentages. To identify discriminative taxa, we applied both the Random Forest classifier and LEfSe (Linear Discriminant Analysis Effect Size), as implemented in MicrobiomeAnalyst. LEfSe was performed with α = 0.05 for the Kruskal–Wallis test and a logarithmic linear discriminant analysis (LDA) threshold of 2.0. A Random Forest classifier was constructed using 500 trees with mtry = 7. Model performance and variable importance (mean decrease in accuracy) were evaluated via Out-of-Bag (OOB) error estimation. This internal validation strategy was adopted to prevent potential data leakage that might occur if patients and their respective cohabiting controls were allocated to different training and testing subsets. Although a household-matching strategy was employed during recruitment to minimize environmental and dietary confounding, data were analyzed using statistical tests for independent samples. Specifically, comparisons between HC and pwRRMS were performed using the Mann–Whitney U test, while the Kruskal–Wallis test was used for multi-group comparisons (e.g., disease severity and disease disability). Paired connections shown in graphical representations were used exclusively for descriptive visualization purposes and to illustrate within-household relationships. No paired statistical tests were applied to these data.

Multiple testing corrections were applied via Bonferroni or Benjamini–Hochberg false discovery rate (FDR), as appropriate. *p*-values are reported as raw (*p*) and adjusted (adj *p*) if they exceed the FDR threshold of 0.05.

## 3. Results

### 3.1. Cohort Description

We enrolled 24 pwRRMS and 24 cohabiting HC (Table 1). All subjects were of Caucasian ethnicity. The ages of groups were similar, whereas, as expected, the MS group had an increased proportion of females (84% compared with 25% in HC). All pwRRMS were under disease-modifying therapies (DMTs) at the time of sampling, which included both low-moderate and highly effective drugs, while none were in relapse at the time of sampling.

Regarding lifestyle habits, no significant differences were observed between groups in terms of smoking status (*p* = 0.3) or childhood exposure to smoke (*p* = 1.0). Individuals with MS reported a lower overall alcohol consumption (*p* = 0.005).

### 3.2. Nutritional Survey

Nutrient intake was assessed using a 7-day self-reported food diary and analyzed with Metadieta^®^ software 4.4 (Table 2). No significant differences were found between groups in total energy, macronutrient, or micronutrient intake. According to Italian national dietary guidelines (LARN) [13], energy intake for both groups fell within the recommended range, with HC closer to the upper limit and pwRRMS nearer the lower end. This aligns with the slightly higher BMI in HC compared to pwRRMS (26.2 vs. 24.0, *p* = 0.11). Protein and fat intake exceeded recommended percentages in both groups, while starch and fiber intake tended to be lower in the MS group (*p* = 0.08).

### 3.3. Gut Microbiota Profiling and Microbial Metabolite Concentration

**Figure 1:**
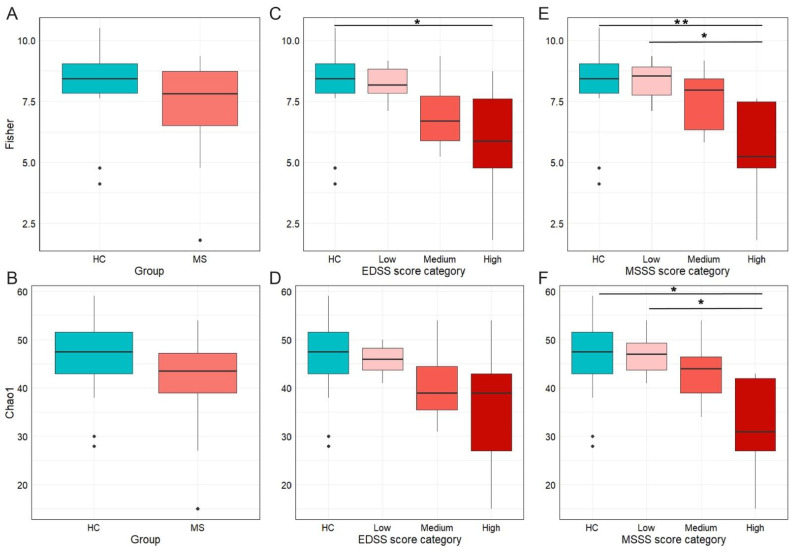
Alpha diversity measures across study groups and clinical score categories. (**A**) boxplots showing the Fisher diversity index by group (healthy controls, HC, vs. persons with multiple sclerosis, MS); (**B**) boxplots showing the Chao1 richness in MS and HC; (**C**) Fisher diversity index according to EDSS score categories; (**D**) Chao1 diversity index according to EDSS; (**E**) Fisher diversity index according to MSSS categories; and (**F**) Chao1 diversity index according to MSSSs. EDSS score categories: low *n* = 12 subjects, medium *n* = 7, high *n* = 5; MSSS categories: low *n* = 8, medium *n* = 11, high *n* = 5. Color intensity increases with disease severity. * *p* < 0.05; ** *p* < 0.01.

Microbiota characterization was performed through V3–V4 16S rRNA gene-targeted sequencing, which yielded a total of 996,310 reads, with an average of 20,595 (max/min 30,356/12,508) reads per sample. α-diversity metrics (Figure 1) revealed a reduced biodiversity in pwRRMS compared to HC. This reduction was statistically significant for the Fisher index (adj *p* = 0.02, Figure 1A), while it did not reach statistical significance for the Chao1 index (adj *p* = 0.06, Figure 1B). Other α-diversity indices—Shannon (adj *p* = 0.13), Simpson (adj *p* = 0.18), and Observed (adj *p* = 0.06)—were not significant. This suggests that the observed decrease in biodiversity is influenced not only by richness but also by community evenness.

To evaluate whether disease disability and disease severity impact the gut microbial community, α-diversity metrics were calculated by stratifying pwRRMS according to EDSS and MSSSs (Figure 1C–F; see Table 1 for detailed severity groups).

Fisher’s index showed a similar distribution across both stratification methods, with progressive decline in microbial biodiversity with increasing disability or rapid progression. A statistically significant reduction in diversity was observed between HC and the high-MSSS group (adj *p* = 0.007, Figure 1E), and a similar trend was noted for the high-EDSS group (adj *p* = 0.051, Figure 1C). No statistically significant differences were detected between HC and either the low-EDSS or low-MSSS groups (adj *p* = 1.000 for both comparisons, Figure 1C,E). The low-MSSS group displayed higher Fisher’s alpha diversity compared with the high-MSSS group (adj *p* = 0.052, Figure 1E). Although other comparisons did not reach statistical significance, they all followed the same downward trend. Chao1 richness estimates followed a comparable pattern, with a progressive reduction in richness from HC to high-disability groups (Figure 1D,F). A significant difference was observed only between HC and the high-MSSS group (adj *p* = 0.018, Figure 1F). Nevertheless, the overall distribution of values reflected a gradual loss of microbial richness with increasing disease severity. Notably, EDSS and MSSSs did not correlate with age (EDSS: Spearman, r = −0.1, adj *p* = 0.6; MSSS: Spearman, r = 0.006, adj *p* = 0.9).

**Figure 2:**
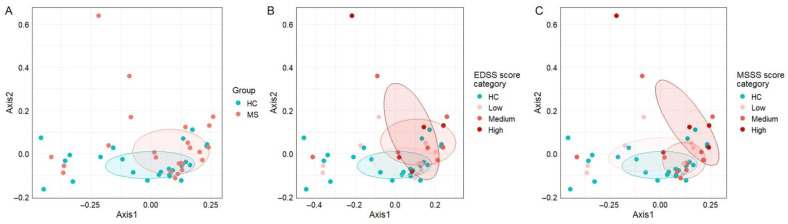
Principal coordinate analysis plots display beta diversity across study groups and clinical score categories. (**A**) HC vs. MS groups. (**B**) Stratification by EDSS score category (low: *n* = 12; medium: *n* = 7; high: *n* = 5). (**C**) Stratification by MSSS category (low: *n* = 8 subjects; medium: *n* = 11; high: *n* = 5).

On the other hand, β-diversity metrics (Figure 2) did not reveal any distinct clustering between the two cohorts (adj *p* = 0.26, Figure 2A). Grouping by EDSS and MSSS categories revealed some visual separation between HC and high-severity groups in both stratifications. A nominal between-group difference was detected between healthy controls and the high MSSS group (*p* = 0.024; adj *p* = 0.144, Figure 2C), indicating a possible trend toward altered microbial composition with greater disease severity. Nevertheless, statistical significance was not maintained after adjustment for multiple comparisons (EDSS: *p* = 0.057; adj *p* = 0.342, Figure 2B).

**Figure 3:**
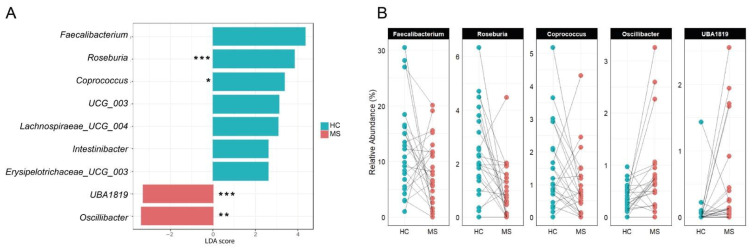
Taxonomy analysis. (**A**) LEfSe analysis identified taxonomic differences in the gut microbiota of subjects with MS (MS) compared with healthy relatives (HC). (**B**) Paired comparison of the relative abundances (%) of *Faecalibacterium*, *Roseburia*, *Coprococcus*, *Oscillibacter*, and *UBA1819* between healthy controls (HC, blue) and persons with multiple sclerosis (MS, red), matched by household cohabitation. * *p* < 0.05; ** *p* < 0.01; *** *p* < 0.001.

The taxonomic analysis showed a significant depletion in pwRRMS of *Roseburia* spp. (1.0% in MS vs. 2.3% in HC; adj *p* = 0.0231) and a trend toward lower abundance of *Intestinibacter* (adj *p* = 0.10) and *Faecalibacterium* (adj *p* = 0.53), though the latter appeared to vary in an individual-dependent manner. Random Forest algorithm was trained and identified *Roseburia* as a biomarker for HC and *UBA1819* as enriched in pwRRMS (Supplementary Figure S1). It should be noted that the Random Forest algorithm was utilized in this study primarily as a robust tool for feature ranking and biomarker discovery rather than as a definitive predictive classifier. Given the inherent heterogeneity of MS and the modest sample size, this approach allowed us to identify key taxa that consistently contributed to group differentiation regardless of the overall model accuracy (OOB error = 0.354, Supplementary Figure S2). These findings were consistent with LEfSE analysis (Figure 3A), which also showed *Oscillibacter* and *UBA1819* enrichment in pwRRMS, while HCs were marked by higher levels of *Roseburia*, *Intestinibacter*, and *Faecalibacterium*.

Figure 3B presents household-matched paired comparisons of key genera identified by LEfSE analysis. *Faecalibacterium* and *Coprococcus* relative abundances were lower in 16 of 24 pwMS (67%), *Roseburia* in 20 of 24 pairs (83%), while both *Oscillibacter* and *UBA1819* showed a higher abundance in 21 of 24 pwMS (88%).

Taxonomy data is extensively detailed in Supplementary Table S1.

**Figure 4:**
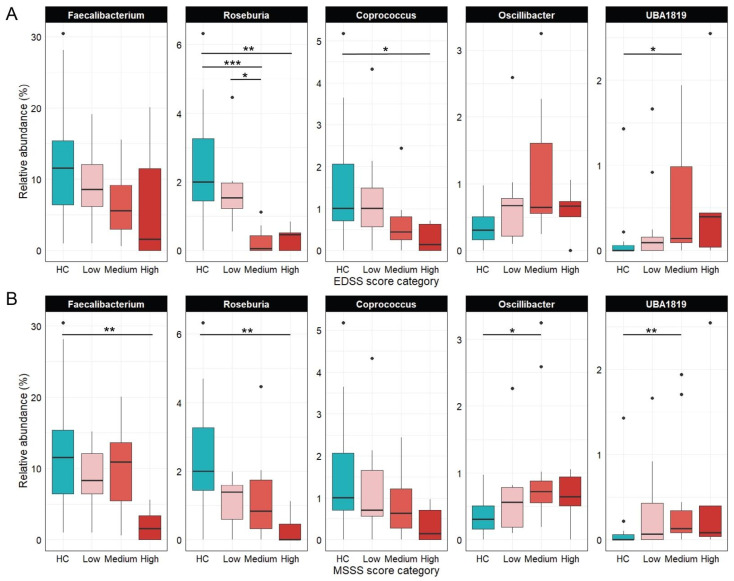
Relative abundance (%) of *Coprococcus*, *Faecalibacterium*, *Oscillibacter*, *Roseburia*, and *UBA1819* in HC and pwRRMS, stratified by EDSS and MSSSs. (**A**) Grouped by EDSS score categories (low: *n* = 12; medium: *n* = 7; high: *n* = 5); (**B**) Grouped by MSSS categories (low: *n* = 8; medium: *n* = 11; high: *n* = 5). * *p* < 0.05; ** *p* < 0.01; *** *p* < 0.001 (adjusted *p*-values).

Figure 4 illustrates shifts in five key genera across EDSS (Figure 4A) and MSSS (Figure 4B) severity categories. *Coprococcus* and *Roseburia* had the highest median abundances in HC and declined with increasing disability. *Coprococcus* was significantly reduced in the high EDSS group (adj *p* = 0.042), while *Roseburia* was markedly lower in the medium (adj *p* = 0.0009) and high EDSS groups (adj *p* = 0.007) compared to controls. *Roseburia* also declined significantly between low and medium EDSS (adj *p* = 0.047) and from HC to the high MSSS group (adj *p* = 0.005), highlighting its inverse association with disease severity. *Faecalibacterium* followed a similar trend but reached significance only in the high MSSS group vs. HC (adj *p* = 0.010). In contrast, *Oscillibacter* and *UBA1819* increased with disease progression: *Oscillibacter* peaked in the medium MSSS group (adj *p* = 0.028), while *UBA1819* was elevated in medium EDSS (adj *p* = 0.016) and MSSS groups (adj *p* = 0.005).

SCFA concentration analysis revealed no statistically significant differences between pwRRMS and HC. Levels of acetic, propionic, and isobutyric acids were comparable in both groups (adj *p* = 0.8285, adj *p* = 0.9260, and adj *p* = 0.9260, respectively), while butyric and isovaleric acids showed lower median values in pwRRMS, though distributions overlapped (adj *p* = 0.4897 and adj *p* = 0.6876, respectively).

**Figure 5:**
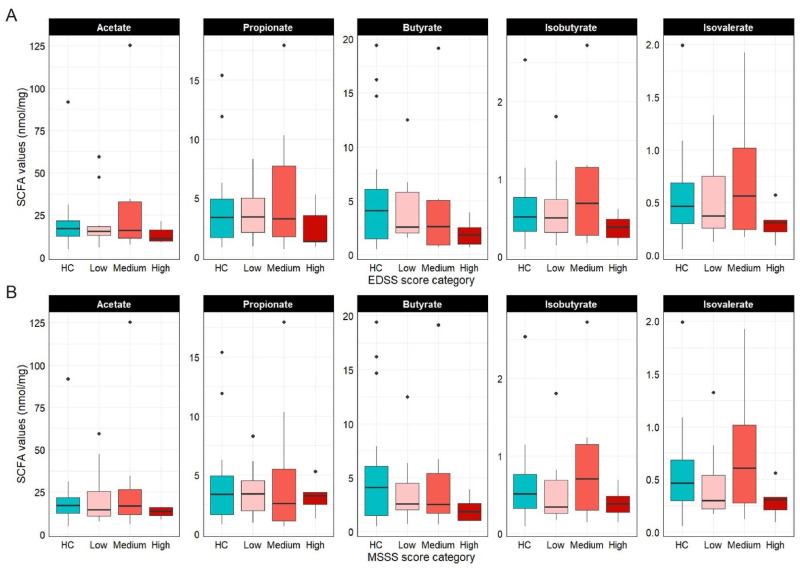
Fecal SCFAs quantification (nmol/mg). (**A**) boxplots showing SCFA levels according to EDSS score categories (low: *n* = 12; medium: *n* = 7; high: *n* = 5); (**B**) SCFA levels according to MSSSs (low: *n* = 8; medium: *n* = 11; high: *n* = 5). Comparisons were not significant.

When stratified by EDSS (Figure 5A) and MSSS (Figure 5B) severity categories, total SCFA levels did not differ significantly across groups. However, a downward trend in median butyrate levels was observed with increasing disease severity, reaching the lowest levels in high-severity patients. Branched SCFAs (isobutyrate, isovalerate) increased in the medium-severity group, followed by a decline in the high-severity category.

## 4. Discussion

An imbalance in the gut microbial composition—commonly referred to as dysbiosis—has been implicated in the development and progression of MS, potentially influencing pathways related to inflammation, neurotoxicity, neurodegeneration, and epigenetic regulation [14].

In this study, we analyzed the gut microbiota composition of 24 individuals with relapsing–remitting multiple sclerosis (pwRRMS) and their household-matched healthy controls. Since both gut microbiota and MS are highly sensitive to environmental influences, distinguishing disease-associated microbial changes from those driven by external factors is challenging. To minimize environmental variability—given the impracticality of fully controlling the living conditions of pwRRMS—we recruited their cohabiting relatives as controls. This strategy, previously validated as effective in reducing environmental noise [15], strengthens the reliability of our microbiota comparisons by accounting for shared lifestyle and environmental exposures, acknowledging that household matching cannot eliminate all possible confounders.

Diet, medication use, and environmental pollutants are key factors shaping gut microbiota composition [16,17], with diet being a primary modulator. Therefore, accounting for dietary intake is crucial when evaluating disease-associated microbial changes. In this study, we assessed the dietary habits of all participants. As anticipated, due to the household-matched design, no significant dietary differences were found between cases and controls, though some deviations from national dietary recommendations were observed.

The gut microbial community was characterized through 16S rRNA gene sequencing, and its core metabolic activity was assessed by quantifying key short-chain and branched-chain fatty acids. Comparisons were first made between HC and pwRRMS, followed by stratification of pwRRMS based on clinical disability and severity scores.

Findings on α-diversity in MS remain inconsistent across studies, reflecting the complexity of microbiome diversity in neuroinflammatory conditions [18]. Our results, partly consistent with previous reports [19], underscore this complexity. When stratified by clinical disability (EDSS) and disease severity (MSSS), healthy controls and patients with low disease burden showed similar α-diversity profiles. In contrast, individuals with severe MS showed a trend toward reduced richness, suggesting a possible link between microbial loss and advanced neurologic impairment. This trend aligns with earlier observations of decreased microbial richness in patients with higher EDSS and MSSSs [20,21], a pattern also seen in other autoimmune diseases, suggesting that a change in GM may be a consequence of MS and autoimmunity [22]. β-diversity analyses revealed no statistically significant community-level differences between MS and HC, with no clear clustering patterns observed. This observation aligns with a subset of previous studies that also failed to detect robust differences in β-diversity metrics between MS and HC [19]. Importantly, recent research has highlighted recruitment site variability as a major confounder in gut microbiota studies [23], potentially obscuring disease-specific microbial signatures. The geographical homogeneity of our cohort likely minimized this confounding factor, strengthening internal validity but possibly limiting the broader applicability of our results.

*Roseburia* emerged as the most disease-affected genus. This anaerobic, Gram-positive keystone taxon is a key butyrate producer and a well-established marker of gut eubiosis. Butyrate serves as a primary energy source for colonocytes and plays a critical role in maintaining intestinal barrier integrity and modulating immune responses. A deficiency in butyrate has been associated with increased intestinal permeability, often referred to as “leaky gut”, a condition implicated in the pathogenesis of MS [24]. Our findings are consistent with previous studies reporting a reduced *Roseburia* abundance in pwRRMS, irrespective of disease activity [19], as well as with data from other inflammatory, autoimmune, and neurological conditions [18]. A parallel decline in other key butyrate-producing genera, such as *Faecalibacterium* and *Coprococcus*, was also observed, consistent with previous findings in pwRRMS [20].

In our cohort, gut microbiota composition showed a tendency to vary with disease severity. Individuals with mild RRMS (low EDSS/MSSSs) displayed microbial profiles broadly comparable to those of healthy controls, whereas greater disability was associated with a trend toward increased dysbiosis. In participants with higher severity scores, we observed a relative depletion of *Roseburia*, *Faecalibacterium*, and *Coprococcus*, alongside a relative enrichment of taxa such as *Oscillibacter* and UBA1819 (family *Ruminococcaceae*). Although these patterns should be interpreted cautiously, given the limited sample size, similar enrichments have been reported in other neurological conditions, including Parkinson’s disease and spinal cord injury, suggesting potential biological relevance [25,26].

Consistent with this pattern, we observed a slight reduction in butyrate and propionate fecal levels in pwRRMS, albeit not at a statistically significant level. This supports the hypothesis that MS-associated microbiome alterations might impair SCFA production and availability, potentially exacerbating inflammation and contributing to disease progression. SCFAs are pivotal mediators of the gut–immune–brain axis. Butyrate and propionate promote the differentiation of regulatory T cells (Tregs) and suppress pro-inflammatory Th1/Th17 responses, thereby shifting immune balance toward an anti-inflammatory phenotype. These metabolites also cross the blood–brain barrier, modulating central nervous system (CNS) immunity [27]. Furthermore, butyrate strengthens both the intestinal and blood–brain barriers by upregulating tight junction proteins and has been shown to promote oligodendrocyte maturation and remyelination in demyelination models [28,29].

While the household-matched case–control design represents a key strength by minimizing environmental variability, it also introduces several limitations. One concern is the imbalance in the male-to-female ratio across cohorts. Although sex-related differences in gut microbiome (GM) composition remain debated [30], the relatively high mean age of participants likely mitigates hormone-driven effects, as sex-associated microbial differences tend to diminish with age [31]. To further assess potential confounding, a sensitivity analysis restricted to the female sub-cohort (*n* = 26) confirmed the significant enrichment of *UBA1819* (*p* = 0.004) and a consistent depletion trend for *Roseburia* (Supplementary Figure S3), supporting the notion that these microbial signatures are primarily linked to MS pathology rather than sex imbalance.

Vitamin D supplementation was also more prevalent in the pwRRMS group compared to healthy controls. Given its known influence on GM composition, potentially promoting beneficial taxa [32], this represents an additional source of confounding. However, both sex distribution and vitamin D intake were highly collinear with disease status in this cohort, making it statistically challenging to disentangle their independent contributions from the effect of MS itself.

Another important limitation is the potential confounding effect of disease-modifying therapies (DMTs), which have been reported to influence GM composition [33]. As all pwRRMS participants were receiving DMT at the time of sampling, it is not possible to fully distinguish microbiome alterations attributable to the disease from those potentially induced or modulated by treatment. These therapies may introduce subtle but meaningful shifts in microbial community structure, potentially obscuring disease-specific signatures [34].

The relatively small sample size further limited the ability to adjust for DMT use and other confounders without compromising statistical power. As a result, subgroup analyses based on EDSS and MSSSs were conducted on small subsets and should be considered exploratory, requiring validation in larger cohorts. Nonetheless, the observed depletion of Roseburia—consistent with findings from independent MS studies—supports the robustness of this result, although confirmation in adequately powered studies remains necessary.

Finally, SCFA quantification represents an additional limitation. Because stool samples were collected directly into a stabilizing buffer without recording initial fecal mass, concentrations could not be expressed in standard units (e.g., µmol/g feces) and were instead normalized to the total stool–buffer mixture. While this approach ensured internal consistency, it may introduce variability due to differences in stool-to-buffer ratios and limit comparability with existing literature. Therefore, SCFA measurements should be interpreted cautiously and considered as relative estimates within this cohort.

## 5. Conclusions

The findings support the presence of gut microbial dysbiosis in individuals with MS, underscoring the potential therapeutic significance of butyrate-producing bacteria and reinforcing the critical role of these bacteria in maintaining gut homeostasis and modulating inflammatory processes. Furthermore, the study demonstrates that a rigorous experimental design can yield statistically robust results even within a limited sample size. This highlights the importance of carefully controlled studies for elucidating the relationships between gut microbiota composition and MS pathogenesis.

## Figures and Tables

**Table 1:** Descriptive statistics of demographic, anthropometric, lifestyle, and clinical characteristics of participants.

	Healthy Controls (*n* = 24)	pwRRMS (*n* = 24)	*p*-Value
**Ethnicity**	Caucasian (24/24)	Caucasian (24/24)	-
**Age, mean ± SD**	48.3 ± 11.1	47.3 ± 11.1	0.757
**Male, *n* (%)**	18 (75)	4 (16)	0.0001
**BMI°, kg/**m^2^	26.1	24.0	0.101
<18.5 (underweight)	0	3	
18.5 < BMI < 24.9 (normal weight)	10	12	
25 < BMI < 30 (overweight)	10	6	
>30 (obesity)	4	3	
Smokers Y/N/P *	5/15/4	5/12/7	0.304
**Childhood Tobacco Exposure**	13/24	13/24	1.0
Alcohol use N/L/M/H **	4/7/6/7	11/6/7/0	0.005
**Vit. D supplementation**	0/24	20/24	-
**Delivery method (vaginal: c-section)**	16:3	17:4	0.79
**Disease duration, mean ± SD**	-	12 ± 10.9	-
**Age at onset, mean ± SD**	-	35.9 ± 15.5	-
**EDSS median (min–max)**	-	2 (0.0–6.0)	-
**Treatment:**	-	24/24	
Azathioprine		1	
Cladribine		1	
Dimethyl Fumarate		6	
Fingolimod		4	
Glatiramer Acetate		1	
Interferon		1	
Natalizumab		2	
Ocrelizumab		1	
Siponimod		1	
Teriflunomide		5	

BMI**°**, body mass index; * Y, yes; N, no; P, past smoker; ** N, no alcohol; L—low-up to 2 drinks/week; M—moderate-3 to 4 drinks/week; H—high-4 drinks/week.

**Table 2:** Daily dietary intake of energy and macronutrients in healthy controls (HC) and individuals with relapsing–remitting multiple sclerosis (pwRRMS).

	HC (Mean ± SD)	pwRRMS (Mean ± SD)	*p*-Value	Reference Values (LARN)
Energy (kcal)	1701.4 ± 497.5	1487.0 ± 332.6	0.0889	1240–1710 kcal/day
Proteins (g)	66.8 ± 20.6	58.9 ± 17.0	0.0851	43–50 (g/day) *
Proteins (%E)	16.3 ± 3.0	16.2 ± 3.4	0.9342	
Fats (g)	69.4 ± 18.4	63.0 ± 15.6	0.3273	
Fats (%)	36.3 ± 8.8	38.8 ± 4.6	0.3172	20–35% E **
Saturated Fats (g)	20.6 ± 8.2	18.2 ± 4.9	0.4393	<10% E
Total Carbohydrates (g)	194.5 ± 76.0	168.1 ± 46.7	0.2699	
Total Carbohydrates (%E)	43.3 ± 5.7	43.0 ± 6.2	0.7105	45–60% E **
Sugars (g)	59.1 ± 38.4	51.3 ± 19.5	0.7966	<15% E
Starch (g)	108.1 ± 43.1	85.4 ± 40.7	0.0889	
Fiber (g)	17.3 ± 10.5	14.5 ± 6.2	0.2835	12.6–16.7 g/1000 kcal
Soluble Fiber (g)	2.5 ± 1.2	1.9 ± 1.1	0.1195	
Insoluble Fibers (g)	8.1 ± 4.5	6.4 ± 3.8	0.1735	

E, Energy; * AR, Average Requirement; ** RI, Recommended Intake.

## Data Availability

The data presented in this study are openly available in the NCBI Short Read Archive (SRA, http://www.ncbi.nlm.nih.gov/sra, accessed on 27 March 2026) under accession number PRJNA1295419.

## Supplementary Materials

The following supporting information can be downloaded at https://www.mdpi.com/2072-6643/18/7/1117/s1, Table S1: Relative abundances of most abundant taxa (≥1%); Figure S1: Top microbial predictors identified by Random Forest analysis. The dot plot displays the most discriminative bacterial taxa ranked by their Mean Decrease Accuracy, representing their relative importance in the classification model; Figure S2: The plot illustrates the error rates of the Random Forest classifier across 500 decision trees using an optimal Feature Set Size (mtry) of 7. The overall Out-of-Bag (OOB) error rate is 0.354, with specific class errors of 0.333 for Healthy Controls (HC) and 0.375 for MS patients (MS). The convergence of the OOB error (red solid line) alongside the class-specific errors (HC, green dashed line; MS, blue dotted line) begins after approximately 200–300 trees; Figure S3: The boxplots represent the filtered and log-transformed counts for the two top predictors within female participants only (HC = 6; MS = 20), showing (A) a trend for *Roseburia* depletion in MS vs. HC although not significant (*p* = 0.15) and (B) confirming UBA1819 enrichment in MS patients (*p* = 0.004).
