# Supplementary material for: Roseburia-Associated Gut–Brain Axis Alterations in Relapsing–Remitting Multiple Sclerosis: Evidence from a Household-Matched Case–Control Study
Source: Nutrients. 2026 Mar 31;18(7):1117. doi: 10.3390/nu18071117 (PMC13074294; doi:10.3390/nu18071117)
Supplement: Supplementary file 1 [file nutrients-18-01117-s001.zip › nutrients-4164790-supplementary.pdf]

Supplementary Table S1 - Relative abundances of most abundant taxa (≥ 1%).

**Phylum**	**Family**	**Genus**	**HC (SD)**	**pwRRMS (SD)**	**p-value (Mann-Whitney)**	**p-adjusted (FDR)**
Actinobacteriota			2.3 (3.0)	4.6 (11.6)	0.6865	
	Bifidobacteriaceae		2.2 (2.8)	4.3 (11.6)	0.3605	
		Bifidobacterium	2.2 (2.8)	4.3 (11.6)	0.3573	0.6970
Bacteroidota			40.4 (11.6)	41.6 (16.5)	0.3409	
	Bacteroidaceae		25.8 (16.1)	28.3 (16.2)	0.5601	
		Bacteroides	25.8 (16.1)	28.3 (16.2)	0.6310	0.9254
		Prevotella_9	7.2 (13.3)	4.7 (12.8)	0.6447	0.9254
	Rikenellaceae		5.1 (4.4)	6.1 (3.9)	0.2911	
		Alistipes	5.1 (4.4)	6.1 (3.9)	0.2911	0.6531
	Prevotellaceae		7.2 (13.3)	4.7 (12.8)	0.6241	
	Tannerellaceae		1.5 (1.1)	1.7 (1.5)	0.7513	
		Parabacteroides	1.5 (1.1)	1.7 (1.5)	0.7648	0.9255
Firmicutes			54.3 (12.1)	50.8 (15.9)	0.1654	
	Eubacterium coprostanoligenes group		2.5 (3.6)	2.3 (3.8)	0.2416	
	Lachnospiraceae		25.4 (6.0)	24.3 (10.5)	0.631	
		Blautia	4.3 (1.8)	5.4 (4.3)	0.6603	0.9254
		Fusicatenibacter	1.7 (1.0)	1.3 (1.1)	0.1406	0.5745
		Roseburia	2.3 (1.5)	1.0 (1.0)	**0.0006**	**0.0249**
		Agathobacter	4.9 (3.5)	4.0 (3.8)	0.2977	0.6531
		Eubacterium hallii group	0.7 (0.5)	1.2 (1.2)	0.1359	0.5745
		Lachnoclostridium	1.2 (0.6)	1.7 (2.5)	0.8933	0.9736
		Lachnospira	1.3 (1.3)	1.4 (2.3)	0.3690	0.6970
		Ruminococcus torques group	1.0 (0.9)	1.1 (1.0)	0.9423	0.9735
	Oscillospiraceae		5.6 (3.7)	5.8 (5.2)	0.7515	
		Subdoligranulum	2.2 (1.9)	2.3 (2.4)	0.5567	0.9234
	Ruminococcaceae		17.0 (8.7)	13.1 (7.2)	0.1305	
		Faecalibacterium	12.2 (7.9)	8.0 (5.9)	*0.0620*	0.5744
		Ruminococcus	1.8 (1.9)	1.5 (2.1)	0.2423	0.6531
		UCG_002	2.7 (2.5)	2.7 (4.4)	0.2662	0.6531
Proteobacteria			2.0 (2.3)	1.7 (1.8)	0.5055	
	Sutterellaceae		1.4 (1.5)	1.3 (1.9)	0.3591	
Verrucomicrobiota			0.7 (2.1)	1.0 (2.0)	0.7356	

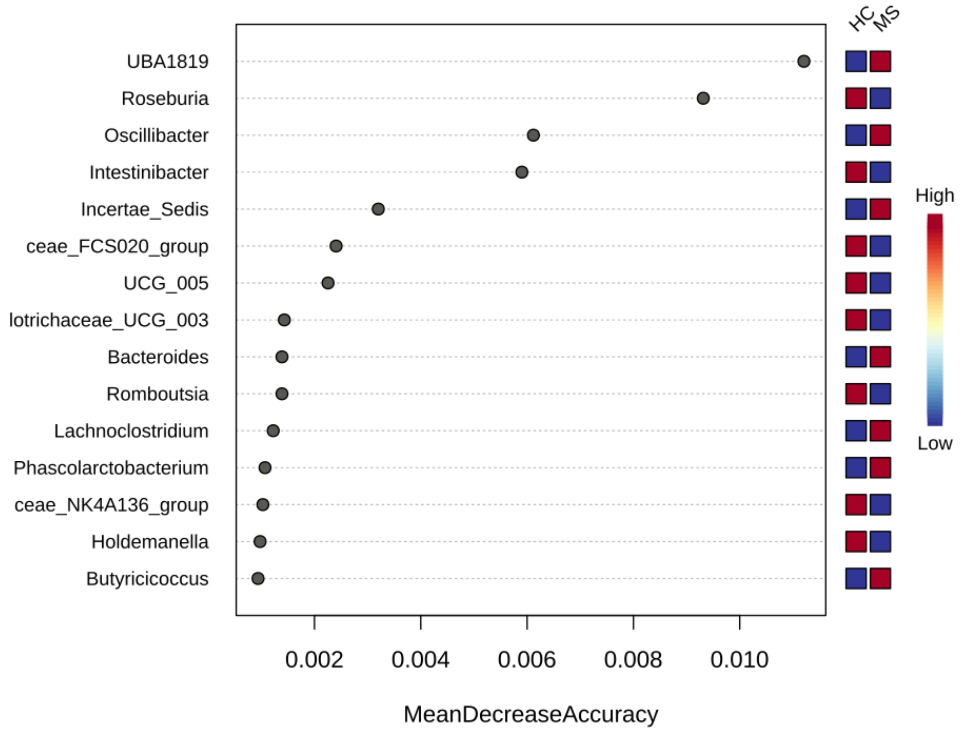

Supplementary Figure S1. Top microbial predictors identified by Random Forest analysis. The dot plot displays the most discriminative bacterial taxa ranked by their Mean Decrease Accuracy, representing their relative importance in the classification model.

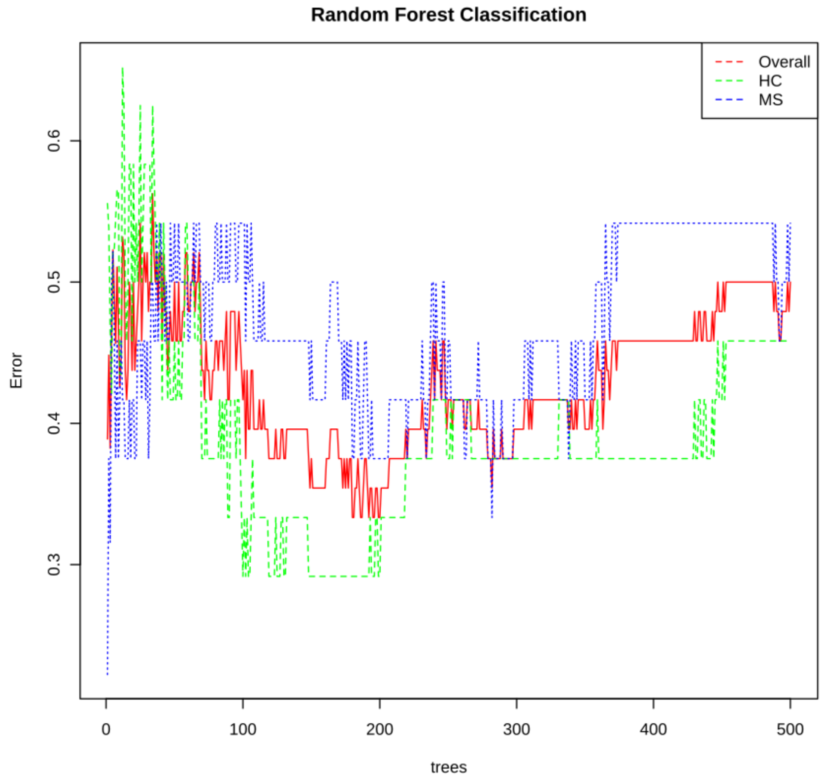

Supplementary Figure S2. The plot illustrates the error rates of the Random Forest classifier across 500 decision trees using an optimal Feature Set Size (mtry) of 7. The overall Out-of-Bag (OOB) error rate is 0.354, with specific class errors of 0.333 for Healthy Controls (HC) and 0.375 for MS patients (MS). The convergence of the OOB error (red solid line) alongside the class-specific errors (HC, green dashed line; MS, blue dotted line) begins after approximately 200–300 trees.

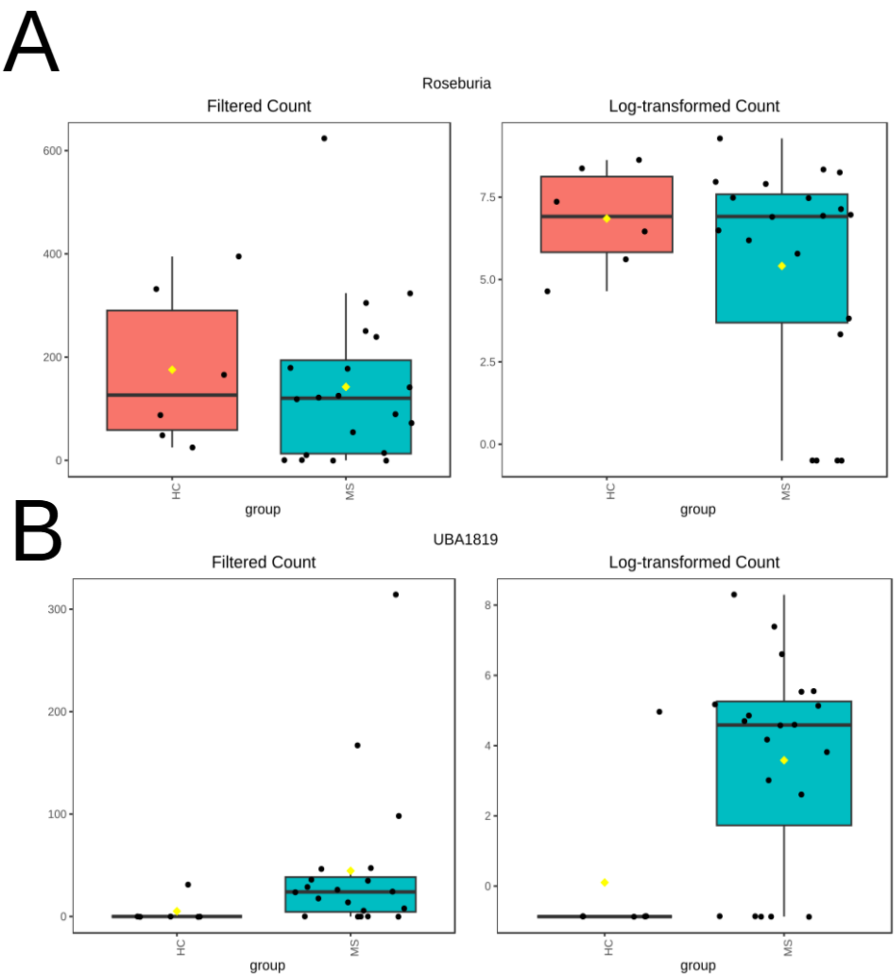

Supplementary Figure S3. The boxplots represent the filtered and log-transformed counts for the two top predictors within female participants only (HC = 6; MS = 20), showing (A) a trend for *Roseburia* depletion in MS vs HC although not significant (p = 0.15) and (B) confirming UBA1819 enrichment in MS patients (p =0.004)
